# Typology and Impact of YouTube Videos Posted in Response to a Student Suicide Crisis: Social Media Metrics and Content Analyses

**DOI:** 10.2196/15551

**Published:** 2021-06-18

**Authors:** Qijin Cheng, Carrie Lui, Flora Wai Lam Ip, Paul Siu Fai Yip

**Affiliations:** 1 Department of Social Work The Chinese University of Hong Kong Hong Kong China (Hong Kong); 2 Hong Kong Jockey Club Centre for Suicide Research and Prevention The University of Hong Kong Hong Kong China (Hong Kong)

**Keywords:** suicide, suicide prevention, social media, infodemiology, internet, digital health, YouTube, impact evaluation, network visualization

## Abstract

**Background:**

Videos relating to suicide are available on YouTube, but their characteristics and impacts have seldom been examined.

**Objective:**

This study aimed to examine YouTube videos posted in response to a sudden spate of student suicides in Hong Kong during the 2015-2016 school year and evaluate the impacts of those videos.

**Methods:**

Keyword search was performed on YouTube, and relevant videos were identified. Video typology was examined through content analysis, specifically grouping the videos by who uploaded the videos, what presentation formats were used in the videos, whether the videos were originally created by the uploaders, and whether the videos disclosed the uploaders’ personal experiences with suicide. Impacts of the videos were assessed in terms of reach (measured by view count), engagement (measured by comment count), and insights (measured as to what extent the comments to each video could reveal personal suicide risk and attitude toward help-seeking). Statistical analysis was conducted to compare the impacts of different types of videos. The 7 most impactful videos that were originally created by the YouTubers were selected for further analysis. They were compared with 7 videos uploaded by the same YouTubers right before the student suicide videos and 7 right after the student suicide videos. The comparison focused on their impacts and the network structure of the comments to those videos.

**Results:**

A total of 162 relevant YouTube videos were identified. They were uploaded by 7 types of stakeholders, and the most common format was one person talking to the camera. A total of 87.0% (141/162) of the videos were originally created by the uploaders and only 8.0% (13/162) of the videos disclosed uploader personal experiences with suicide. The uploader profiles being popular or top YouTubers and the video containing disclosure of the uploader’s personal experiences were found to be significantly correlated with greater impacts (*P*<.001). Focusing on the 7 most impactful original videos, it is found that those videos generated more engagement, especially more interactions between the viewers, and more insights than regular videos uploaded by the same YouTubers.

**Conclusions:**

When responding to a youth suicide crisis, videos made by key opinion leaders on YouTube sharing their own experiences of overcoming suicide risks could generate significant positive impacts. These types of videos offer a precious opportunity to craft online campaigns and activities to raise suicide prevention awareness and engage vulnerable youth.

## Introduction

Youth suicide is an alarming public health problem. Suicide is the second leading cause of death among young people aged 15 to 24 years globally and accounts for over 16% of all deaths among youth [1]. Social media is a ubiquitous form of communication for young people nowadays, and this has become a double-edged sword for suicide prevention [2-5]. On one hand, social media may increase the availability and accessibility of suicide method information or connect suicidal people to form suicide pacts. On the other hand, social media can also offer a new channel for raising public awareness and for suicidal people to disclose their feelings and look for help [6]. Making good use of social media to engage at-risk youngsters and connect them with preventive resources is imperative for youth suicide prevention.

YouTube, a video-sharing site which has over a billion users globally, is one of the most popular social media sites [7]. A couple recent studies used keyword search to identify YouTube videos about suicide in English or German and conducted content analyses on the most-viewed videos or a random sample of the returned videos [8,9]. One study applied existing media guidelines of responsibly reporting suicide to evaluate the appropriateness of suicide-related YouTube videos in the German language and found that those videos exhibited more potentially harmful than protective characteristics [8]. However, the other study applied the open coding method to summarize the content themes exhibited in YouTube videos about teen suicide in English and found that the majority of them were educational or raised awareness of suicide prevention [9]. Another approach is to assess the impacts of a specific YouTube video using social media metrics [10]. The commonly evaluated metrics include: (1) reach (ie, number of views); (2) engagement (ie, number of comments or responses); and (3) insights (ie, whether the audience engagement is positive, neutral, or negative) [11]. Furthermore, a study has applied a machine learning method to automatically identify YouTube comments in the Traditional Chinese language that showed suicide risk for further intervention [12]. Nonetheless, compared to other online media, such as search engines, online forums, Facebook, Twitter, and Weibo (a Chinese social media), YouTube has been the focus of relatively fewer studies as related to suicide.

In the 2015-2016 school year (from September 1 to August 31), 33 students unfortunately died by suicide in Hong Kong, of which the majority were teenagers and the most common method was jumping from height [13,14]. The significant increase of incidents was alarming when compared to 19 student suicides in each of the 2013-2014 and 2014-2015 school years [14]. When 11 student suicides occurred during the period between February 12 and March 28, 2016, the seeming cluster triggered intense media coverage and online discussion. To curb the crisis of student suicides, the Hong Kong government set up a committee on student suicide prevention on March 30, 2016, constituting public health experts, psychiatrists, psychologists, social workers, school principals and teachers, parents, youth leaders, and government officials. Responding to the committee’s advice, local press reduced sensational reporting of student suicides and published more preventive information, which was found to be associated with a subsequent decrease in student suicides toward the end of 2016 [13]. During the process, various community members spontaneously made use of YouTube to voice their thoughts and opinions regarding student suicides.

The series of events, which will be referred as the student suicide crisis hereafter, offered a precious opportunity for us to observe what role YouTube can play when a youth suicide crisis is in the spotlight. Combining the approaches of both content analysis and impact assessment, this study specifically examined what YouTube videos were posted in response to the student suicide issue in Hong Kong and what impacts those videos generated. Specifically, our research questions include the following:

- What kinds of videos are publicly accessible on YouTube regarding the student suicide crisis?
- How much impact did different kinds of videos generate in terms of reach, engagement, and insights?
- Which types of videos were more likely to generate greater impacts?

As the study was exploratory and data-driven in nature and there was no previous study on the same topic, we had no hypothesis regarding those questions.

## Methods

### Data Collection

**Figure 1:**
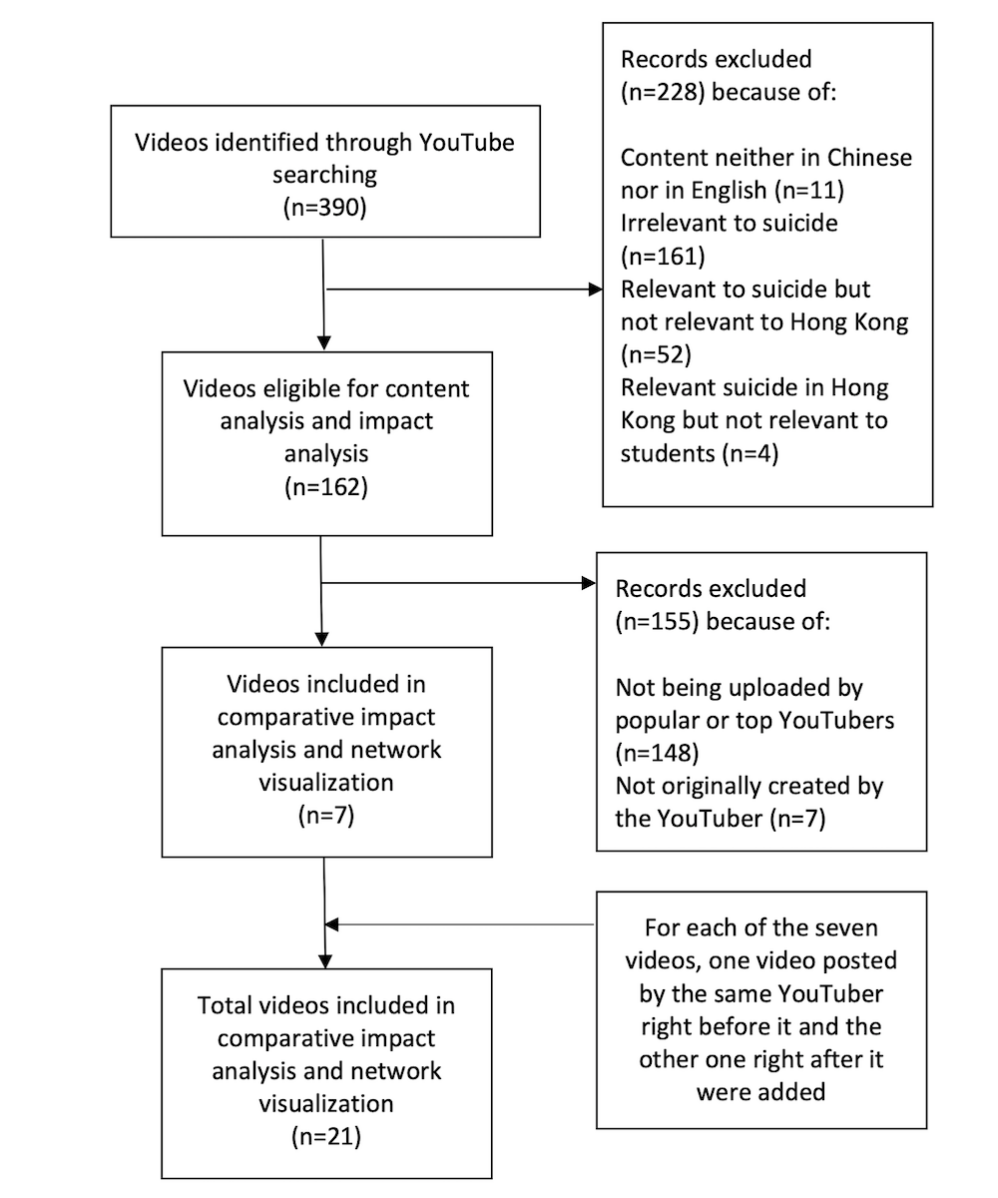
Procedure of video searching, screening, and analysis.

The data collection procedure is illustrated in Figure 1. Using YouTube’s open application programming interface, videos were searched and downloaded with the Traditional Chinese keywords relating to student suicides that were frequently being used in local newspapers when reporting the student suicide crisis. The query was written as [學生自殺 (student suicide), 學童自殺 (school children suicide) OR 學生跳樓 (student jumping from height) OR 學業壓力 (academic pressure) OR 珍惜生命 (cherish life)]. The search period was restricted to the 2015-2016 school year (ie, from September 1, 2015, to August 31, 2016). Data such as the title and description of each video, URL, uploader’s user names and self-report profiles, upload date, as well as the numbers of views, likes, dislikes, comments, and replies were downloaded up until June 8, 2017. Readers are reminded that there are two layers of comments on YouTube, of which the first layer are the comments directly address to the video and the second layer are the replies to some of the first layer comments. YouTube counts the total number from both layers when reporting the number of comments. To protect YouTube users’ privacy, their user names were replaced by nonidentical numbers in this paper.

The search initially yielded 390 videos. Each video was assessed on its relevance with the student suicide crisis and only videos meeting the following criteria were eligible for further review: the video must be presented in the Chinese language, either Cantonese or Mandarin, and the video’s content should be reporting or addressing the series of student suicides that happened during the specified period in Hong Kong. A total of 162 videos were found to be eligible for further analyses. The full-text content of the comments and replies to the 162 videos and the user IDs of those who posted comments and replies were then downloaded.

After we analyzed the impact metrics of the eligible videos, 7 videos were identified as the most impactful. An additional analysis on the impacts of the 7 videos was conducted. For each of the 7 videos (hereinafter referred as the student suicide videos), one video posted by the same YouTuber right before the incident video (hereinafter referred as the prevideo) and another video right after the incident video (hereinafter referred as the postvideo) were identified and the metadata of the comments and replies were downloaded. By comparing the impact metrics of the student suicide video with the impact metrics of the same YouTuber’s prevideos and postvideos, we aimed to identify special impacts of the student suicide videos by controlling the uploaders’ profiles.

### Content Analyses

The content of the videos and video uploader profiles as described in the About section of the 162 relevant videos were reviewed. Two authors (QC and FI) examined the 162 videos together and categorized the 162 video uploaders and video format by intensive discussions. The two authors reached a full consensus on the final coding scheme and results.

Video uploaders were first differentiated as either an organization or an individual, according to the descriptions in their About Us page. Furthermore, they were categorized into 7 groups: (1) traditional media (media outlets that operate not only YouTube channels but also publish offline formats such as newspapers, TV, or radio channels); (2) online organizations (media or professional organizations that only publish content online); (3) government bodies, which include the Hong Kong government’s information department and the police force; (4) politicians, who are mostly local legislators; (5) top YouTubers (individuals operating YouTube channels with 100,000 or more subscribers); (6) popular YouTubers (individuals operating YouTube channels with 10,000 to 100,000 subscribers); and (7) regular YouTubers (individuals operating YouTube channels with fewer than 10,000 subscribers). The first 3 categories were all self-presented as organizations, whereas the other 4 were all individuals.

The video formats were categorized into 4 groups: (1) simple speech, which is one person talking in front of a camera; (2) conversations, which are often talk shows, panel discussions, or debates at legislative meetings; (3) news or documentaries, which are often news reports or feature reports produced by media professionals; and (4) fictional performances, which include one short film created by a group of students and a music video. Each video was assigned to only one type of uploader and one type of video format. In addition, whether a video was originally created by the uploader and whether the video disclosed the uploader’s personal experiences with suicidal thoughts or behaviors were also marked.

As for the comments, the two authors first randomly selected 100 comments and individually conducted open coding on their content, focusing on the impact measurements. The two then compared their codes and discussed their interpretations. Based on their discussions, a coding scheme was generated. The scheme categorized 3 common themes from the comments: displaying positive or negative attitudes toward the subject video, disclosing that the commenter was having or has had suicidal thoughts or behaviors before, and showing supportive or discouraging tendencies toward help-seeking. For theme 3, comments showing supportive tendencies to help-seeking included those showing willingness to seek help, showing support to people who needed help, and sharing experiences of how they received help from others. Comments showing discouraging tendencies to help-seeking were those indicating that existing help resources were useless. Another 100 comments were randomly selected and the two authors individually annotated whether a comment exhibiting one of the 3 themes. The interrater reliability between the two authors was then examined by Krippendorff alpha and the result was .94, indicating high reliability [15]. Thereafter, one of the two authors (FI) completed the coding of all comments following the coding scheme.

### Statistical Analyses

Among the 3 impact metrics, reach and engagement were measured by the numbers of views and comments, which were directly extracted from the YouTube archive data. Insights were measured by counting how many comments showed a theme identified by the content analysis. Comparing different categories of video impacts, a Kruskal-Wallis rank sum test was employed to examine the variance due to nonnormality of the impact metrics. All statistical analyses were performed using SPSS (version 23, IBM Corp).

During the data analysis, we noted that 7 videos that were uploaded by popular and top YouTubers who disclosed their personal experiences with suicide generated significantly greater impacts than other videos. In view of their special influence, a further analysis focusing on the 7 videos was conducted to rule out the possible effects from their large number of subscribers on the impact metrics. Specifically, we compared the 7 student suicide videos with their corresponding prevideos and postvideos made by the same 7 YouTubers. Descriptive statistics were used to quantify the number of total comments and the number of unique users who only commented on the student suicide videos but not on the prevideos and postvideos. Furthermore, the lengths of comments (ie, number of words) were compared between the student suicide prevention videos and the prevideos and postvideos by *t* test.

Network visualization was also used to compare the interaction network structures between the student suicide videos and the prevideos and postvideos. Based on the comment data, the network graph depicts each unique user ID as a node and a comment as a directed edge from who posted the comment to whom the comment was addressed. Three network graphs were produced to illustrate the commenting structures at the moment of the prevideos, the student suicide videos, and the postvideos. The network graphs were visualized with the open-source software Gephi.

## Results

### Typology of the Videos

As shown in Table 1, the majority of the videos were uploaded by traditional media (52/162, 32.1%) and online organizations (44/162, 27.2%), followed by regular YouTubers (35/162, 21.6%), politicians (12/162, 7.4%), popular YouTubers (10/162, 6.2%), government bodies (5/162, 3.1%), and top YouTubers (4/162, 2.5%). All videos showed a general tendency to support suicide prevention, although they had varied views on what prevention measures might be effective or ineffective. Most of the videos were shot in a simple speech format (ie, one person talking in front of a camera; 82/162, 50.6%), followed by professionally edited news clips or documentaries (56/162, 34.6%) and conversations between multiple people (22/162, 13.6%). Only two videos (2/162, 1.2%) were fictional performances. About 87% (141/162) were originally created by the uploaders, with 8% (13/162) disclosing personal experiences on how to battle with suicidal thoughts or behaviors. The remaining 13% (21/162) were news clips or video records of legislative meetings or other public activities created by the uploaders.

**Table 1:** Categories of YouTube videos responding to the student suicide issue and a comparison on their reach and engagement (n=162).

Category			Videos, n		Number of views							Number of comments				
					Median (range)		Mean rank		Chi-square (*P* value)		Median (range)			Mean rank		Chi-square (*P* value)
By uploader profile			—^a^		—		—		39.3 (<.001)		—			—		36.6 (<.001)
	Traditional media	52		1700 (51-45,143)		97.31		—		.75 (0-45)			86.66		—	
	Online organizations	44		476 (7-41,237)		79.56		—		.28 (0-34)			67.22		—	
	Regular YouTubers	35		151 (22-373,478)		53.46		—		.39 (0-104)			72.87		—	
	Politicians	12		275 (22-22,712)		61.63		—		.56 (0-21)			79.71		—	
	Popular YouTubers	10		5784 (121-16,015)		115.10		—		18.50 (0-229)			128.40		—	
	Government bodies	5		148 (71-698)		48.60		—		.20 (0-1)			61.20		—	
	Top YouTubers	4		97,215 (72,049-13,1596)		159.50		—		965.50 (402-1582)			160.50		—	
By video format			—		—		—		5.3 (.15)		—			—		4.6 (.20)
	One person talking	82		351.50 (22-373,478)		74.80		—		.53 (0-1582)			81.52		—	
	News or documentary	56		1308 (44-45,143)		93.04		—		.71 (0-45)			85.40		—	
	Two or more people talking	22		489.50 (7-6280)		76.55		—		.28 (0-24)			68.00		—	
	Fictional performance	2		1266.50 (281-2252)		87.75		—		5.00 (1-9)			119.75		—	
Original creation			—		—		—		0.004 (.95)		—			—		.10 (.75)
	Yes	141		499 (7-131,596)		81.41		—		.56 (0-104)			81.11		—	
	No	21		536 (27-343,478)		82.10		—		.57 (0-1582)			84.14		—	
Disclosure of personal experience			—		—		—		9.4 (.002)		—			—		11.9 (.001)
	Yes	13		5764 (81-121,719)		119.69		—		4.33 (0-1260)			119.35		—	
	No	149		433 (7-343,478)		78.17		—		.48 (0-1582)			78.20		—	

^a^Not applicable.

### Relationships Between Video Characteristics and Video Impacts

Table 1 and Multimedia Appendix 1 compared different types of video impacts. Results showed that disclosing uploader’s personal experience with suicide was significantly correlated with greater impacts in all dimensions except for the number of comments criticizing the video and the number of comments negative to help seeking. In other words, videos with this feature demonstrated greater positive impacts than those without it, but there was no significant difference from those without it in terms of negative impacts.

Uploader profiles were found to be another factor related to the video impacts. Pairwise post hoc test of Kruskal-Wallis rank sum test (see details in Multimedia Appendix 2) found that, in terms of reach, top YouTubers outperformed politicians, government bodies, and regular YouTubers but not popular YouTubers, traditional media, or online organizations. This might be attributed to that fact the YouTube channels of popular YouTubers, traditional media, and online organizations also have substantial numbers of subscribers, which laid a foundation for higher view counts. In terms of engagement, top YouTubers further outperformed traditional media and online organizations, and only popular YouTubers reported comparable numbers of comments with top YouTubers. Among the 7 types of uploaders, government body videos received the smallest numbers of views (median 148, maximum 698) and comments (median 0.20, maximum 1). In terms of insights, top YouTubers even significantly outperformed popular YouTubers except for the number of comments showing negative attitude toward help-seeking. In summary, although those uploaders with larger numbers of subscribers mostly generated greater reach, top and popular YouTuber videos were more engaging, and top YouTuber videos were able to stimulate more insightful comments. This phenomenon might be explained by the disclosing of the uploaders’ personal experiences with suicide because most of the student suicide videos uploaded by the top YouTubers mentioned their own suicidal thoughts when they were students and how they overcame the thoughts by getting help from their friends or family members. In contrast, half of the videos uploaded by popular YouTubers were reposting news clips or just sharing other people’s experiences of suicidal thoughts.

Informed by the results, we decided to focus on the 7 videos uploaded by top and popular YouTubers (referred as KOLs, or key opinion leaders) who disclosed their personal experiences with suicide to conduct additional analysis on their impacts.

### KOL Student Suicide Videos Versus Their Regular Videos

The 7 selected videos were uploaded by 7 different YouTubers. For each of the videos, one prevideo and one postvideo uploaded by the same YouTuber were included for the comparison. The prevideos and postvideos were found to be related to internet gaming, traveling, or other entertainment topics, which are regular trendy content created by those KOLs.

**Figure 2:**
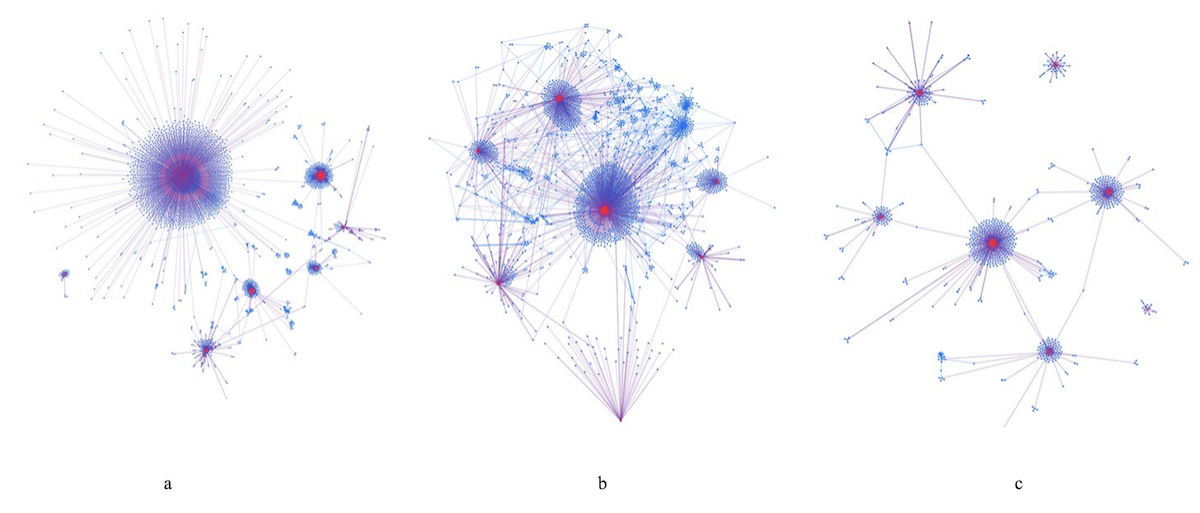
Network graph comparison of the comments to the 7 most impactful student suicide videos with their pre- and post-videos uploaded by the same YouTubers: (a) comments to the pre-videos (2187 nodes, 2398 edges), (b) comments to the student suicide-related videos (2175 nodes, 2816 edges), and (c) comments to the post-videos (721 nodes, 825 edges). Notes: Red nodes denote video uploaders and blue nodes denote users who posted comments. Purple edges denote first layer comments from commenters to the video uploaders and blue edges denote replies from commenters to commenter.

As shown in Multimedia Appendix 3, compared with the prevideos and postvideos, the student suicide videos received fewer view counts but attracted more and longer comments. Those KOLs also had been more active in replying to their viewer comments regarding the student suicide videos (29 vs 60 replies) than their regular videos (5 vs 33 replies). The only exception was one top YouTuber’s prevideo (ie, Top 2 in Multimedia Appendix 3), in which a new video game was introduced, that received more comments than his student suicide video (1946 vs 1259). However, his prevideo received fewer second layer replies than his student suicide video (161 vs 404). In addition, his prevideo’s first layer comments and second layer replies were both significantly shorter than his student suicide video’s (*P*<.001; see details in Multimedia Appendix 3). The results suggested that even with the same influential YouTubers, their student suicide videos could generate more in-depth interactions among their viewers. Multimedia Appendix 3 also demonstrates that 83.1% (133/160) to 96.8% (637/658) of the users who commented on the student suicide videos did not post any comments on the same YouTuber’s prevideos and postvideos, suggesting that they may not be actively engaged by those regular videos. Figure 2 illustrates the social network structures of the comments. The prevideo and postvideo comments network figures (Figure 2a and 2c) were rather similar. They both had 7 centralized clusters surrounding the 7 KOLs, and both were dominated by purple edges, reflecting that regular viewers were generally commenting to the KOLs. The 7 clusters were not well connected with each other, if not at all, suggesting little overlapping between users who commented to different KOLs’ regular videos. By contrast, blue edges in Figure 2b were more noticeable, indicating more interactions among regular viewers which distracted the KOLs’ centralization role. In addition, the 7 clusters became better connected with each other directly or indirectly in Figure 2b.

## Discussion

### Principal Findings

To the best of our knowledge, this study is the first to examine the typology and impact metrics of YouTube videos responding to a student suicide crisis. When a spate of student suicides occurred in Hong Kong, various community members voiced their concerns through YouTube videos and, fortunately, we found the videos were generally supportive of suicide prevention and encouraged young people to cherish their lives. The results highlighted the important role of YouTube KOLs, including popular and top YouTubers who create their own original content, in suicide prevention. Although other YouTube channels with large numbers of subscribers, such as traditional media and online organizations, could also achieve a substantial reach, KOL videos, especially those disclosing their own experiences with overcoming suicidal thoughts, exhibited more outstanding performance in terms of engagement and insights. Those videos might have resonated with viewers and validated their feelings more, especially for those who also had suicidal thoughts. In addition, there were more peer interactions in the comments to those KOL student suicide videos, which might have made the viewers feel less lonely and encouraged further disclosure of their experiences and feelings. The findings add new evidence to the potential positive effects of social media when the media content is about suicide prevention [13,16,17]. It is noted that popular YouTubers can achieve comparable impacts to top YouTubers in most of the dimensions. Therefore, when there is a dearth of top YouTubers and they are too costly to be partner with, we could engage popular YouTubers on a larger scale as an alternative to achieve desirable impacts.

This study found that the local government bodies and professional organizations did release suicide prevention videos on YouTube addressing the student suicide crisis, but their impacts were far smaller than the KOL videos. Notably, the 14 videos uploaded by top and popular YouTubers generated significantly more views and comments than the 49 videos uploaded by government bodies and online organizations (465,596 views and 4509 comments vs 206,630 views and 86 comments). The findings suggests that, if professional organizations and government bodies would like to make use of YouTube to raise awareness of suicide prevention among young people and engage vulnerable youth, they might be more effective by leveraging KOL influences and life experiences to create videos rather than producing their own videos. Although not captured in this study, we should be aware that occasional incidents of YouTube videos showing suicide scenes or the process of suicidal behaviors or containing tips on how to commit suicide have been reported by the news media. Therefore, suicide prevention professionals are advised to have in-depth discussions with KOLs to understand their background and views on suicide and suicide prevention, if a formal collaboration is going to be established.

Comparing the same KOLs’ regular videos, their student suicide videos were particularly engaging in terms of the number and length of comments and the interaction among the audience. The finding could encourage KOLs to produce more of these kinds of videos because it would be beneficial for them to engage their followers and form a healthy and caring online community.

Meanwhile, the findings also deserve suicide prevention professionals’ attention and follow-up. The comments offered valuable insights to uncover at-risk individuals, discern what people appreciate or dislike in a suicide prevention video and why people at risk would seek help or not. Even though some comments were negative or discouraging to help-seeking, they have provided critical feedback for future improvements of social support in society. Furthermore, in view of some KOLs’ genuine interests in suicide prevention, gatekeeper training and support should be provided to them to empower them with the appropriate skills to communicate with their followers who are at risk of suicide and refer those in need of professional care and interventions. As disclosed in the comments of the student suicide videos, there were users who were at risk of suicide or indicated willingness to help with suicide prevention activities. For the former, suicide prevention professionals can work with the KOLs to engage vulnerable individuals and refer them to suicide prevention services such as online 24/7 emotional support services [18]. For the latter, they can be engaged as gatekeepers to promote suicide prevention in the online social network or within their own social circles. It would be helpful if a connected care model can be established to facilitate online gatekeepers to refer vulnerable youth to offline professional services.

### Limitations

A few limitations of the study should be noted. First, some relevant videos may not be included in the study if those videos did not explicitly include the keywords that we employed or were uploaded in the private domain. There might also be private communications between the YouTube users that could not be captured in this study. Second, since the reviewed videos were posted on different dates, direct comparison of the number of views and comments of the videos was seemingly unfair. Nonetheless, the vast majority of views and comments on the YouTube videos were received on the first week after release, and the data collection date in this study was at least 5 months after the release dates of those videos. This can minimize the bias in the discrepancies of the data. The cutoff of subscriber numbers for defining popular and top YouTubers in this study was adopted from the common practice in the marketing industry in Hong Kong in 2017. As the number of users of YouTube varies in different contexts, the cutoff of popular and top YouTubers is also context-sensitive. Other researchers need to check the up-to-date marketing reference to identify KOLs in their own context.

### Conclusion

Youth suicide prevention is a global challenge. During the difficult times of COVID-19, the risk of suicide is also increasing [19]. This study demonstrates that, when responding to a public crisis of youth suicide, videos made by YouTube KOLs sharing their own experiences of overcoming suicide risks could generate significant positive impacts. Suicide prevention professional and government bodies should take the opportunity to collaborate with those KOLs to deliver online campaigns and interventions to raise awareness of suicide prevention and engage vulnerable youth. Also, the KOLs themselves can seize the opportunity to provide support for their followers. Participating in promoting mental wellness would likely enhance their public image and reachability to the social media users. In the meantime, more empirical evaluations of this type of videos are imperative to further identify effective measures that can make the best of social media for suicide prevention.

## Appendix

Multimedia Appendix 1 Comparison of the insights different types of videos generated.

Multimedia Appendix 2 Pairwise post hoc tests on the relationships between different types of videos and their impacts.

Multimedia Appendix 3 Comparing the top 7 impactful student suicide-related videos with their pre- and post-videos uploaded by the same YouTubers.

## Abbreviations

KOL: key opinion leader
