# Supplementary material for: Typology and Impact of YouTube Videos Posted in Response to a Student Suicide Crisis: Social Media Metrics and Content Analyses
Source: JMIR Ment Health. 2021 Jun 18;8(6):e15551. doi: 10.2196/15551 (PMC8277376; doi:10.2196/15551)
Supplement: Multimedia Appendix 1 [file mental_v8i6e15551_app1.docx]

**Multimedia Appendix 1. Comparison on the insights that different types of videos generated.**

|  | **No. of videos** | **No. of Comments supporting the video** | | | **No. of Comments criticizing the video** | | | **No. of Comments positive to help seeking** | | | **No. of Comments negative to help seeking** | | | **No. of Comments disclosing suicide risk** | | |
| --- | --- | --- | --- | --- | --- | --- | --- | --- | --- | --- | --- | --- | --- | --- | --- | --- |
|  |  | **Median (range)** | **Mean Rank** | **Chi-Square (***P*-value**)** | **Median (range)** | **Mean Rank** | **Chi-Square (***P*-value**)** | **Median (range)** | **Mean Rank** | **Chi-Square (***P*-value**)** | **Median (range)** | **Mean Rank** | **Chi-Square (***P*-value**)** | **Median (range)** | **Mean Rank** | **Chi-Square (***P*-value**)** |
| ***By Uploader’s Profile*** | | | | | | | | | | | | | | | | |
| **Traditional media** | 52 | .08 (0 ~ 3) | 78.85 | 53.377 (<.001) | .06 (0 ~ 1) | 75.91 | 39.360 (<.001) | .06 (0 ~ 6) | 79..53 | 80.875 (<.001) | .04 (0 ~ 4) | 81.13 | 29.604 (<.001) | .10 (0 ~ 1) | 81.36 | 62.565 (<.001) |
| **Online organizations** | 44 | .05 (0 ~ 4) | 76.53 |  | .12 (0 ~ 5) | 80.57 |  | .00 (0 ~ 0) | 75.00 |  | .02 (0 ~ 1) | 79.81 |  | .02 (0 ~ 1) | 75.74 |  |
| **Regular YouTubers** | 35 | .12 (0 ~ 15) | 77.60 |  | .12 (0 ~ 6) | 80.73 |  | .03 (0 ~ 1) | 77.17 |  | .00 (0 ~ 0) | 78.00 |  | .06 (0 ~ 2) | 76.30 |  |
| **Politicians** | 12 | .08 (0 ~ 1) | 79.25 |  | .08 (0 ~ 1) | 77.88 |  | .00 (0 ~ 0) | 75.00 |  | .00 (0 ~ 0) | 78.00 |  | .00 (0 ~ 0) | 74.00 |  |
| **Popular YouTubers** | 10 | 0 (0 ~ 45) | 106.35 |  | 0 (0 ~ 12) | 95.80 |  | 0.5 (0 ~ 35) | 115.05 |  | 0 (0 ~ 1) | 93.90 |  | 0 (0 ~ 13) | 106.95 |  |
| **Government bodies** | 5 | .00 (0 ~ 0) | 73.00 |  | .00 (0 ~ 0) | 71.50 |  | .00 (0 ~ 0) | 75.00 |  | .00 (0 ~ 0) | 78.00 |  | .00 (0 ~ 0) | 74.00 |  |
| **Top YouTubers** | 4 | 199.50 (27 ~ 373) | 160.00 |  | 7.5 (4 ~ 12) | 158.75 |  | 72 (14 ~ 104) | 160.25 |  | 1 (0 ~ 10) | 119.50 |  | 62.5 (18 ~ 71) | 160.50 |  |
| ***By Video Format*** | | | | | | | | | | | | | | | | |
| **One person talking** | 82 | .16 (0 ~ 373) | 85.04 | 7.464 (.058) | .20 (0 ~ 12) | 86.43 | 5.950 (.114) | .12 (0 ~ 104) | 84.01 | 2.224 (.527) | .06 (0 ~ 10) | 82.93 | 1.767 (.622) | .21 (0 ~ 71) | 82.24 | 4.108 (.250) |
| **Two or more people talking** | 22 | .18 (0 ~ 4) | 76.66 |  | .10 (0 ~ 12) | 79.07 |  | .09 (0 ~ 2) | 78.57 |  | .00 (0 ~ 0) | 78.00 |  | .09 (0 ~ 2) | 77.66 |  |
| **News or documentary** | 56 | .05 (0 ~ 3) | 77.09 |  | .05 (0 ~ 1) | 75.60 |  | .06 (0 ~ 6) | 79.21 |  | .04 (0 ~ 4) | 80.90 |  | .09 (0 ~ 1) | 80.83 |  |
| **Fictional performance** | 2 | 2.00 (0 ~ 4) | 113.25 |  | .00 (0 ~ 1) | 71.50 |  | .00 (0 ~ 0) | 75.00 |  | .00 (0 ~ 0) | 78.00 |  | .50 (0 ~ 1) | 112.25 |  |
| ***Original Creation*** | | | | | | | | | | | | | | | | |
| **Yes** | 141 | .12 (0 ~ 373) | 82.18 | .802 (.371) | .13 (0 ~ 12) | 81.19 | .148 (.701) | .09 (0 ~ 104) | 81.93 | .410 (.522) | .05 (0 ~ 10) | 82.02 | 1.082 (.298) | .11 (0 ~ 71) | 82.05 | .583 (.445) |
| **No** | 21 | .71 (0 ~ 15) | 76.95 |  | .16 (0 ~ 12) | 83.60 |  | .05 (0 ~ 1) | 78.62 |  | .00 (0 ~ 0) | 78.00 |  | .10 (0 ~ 2) | 77.83 |  |
| ***Disclosure of Personal Experience*** | | | | | | | | | | | | | | | | |
| **Yes** | 13 | .56 (0 ~ 237) | 104.81 | 12.334 (<.001) | .27 (0 ~ 12) | 90.92 | 1.748 (.186) | .80 (0 ~ 93) | 100.19 | 10.112 (.001) | .77 (0 ~ 10) | 84.46 | .454 (.500) | .80 (0 ~ 71) | 99.81 | 8.516 (.004) |
| **No** | 149 | .09 (0 ~ 373) | 79.47 |  | .12 (0 ~ 12) | 80.68 |  | .06 (0 ~ 104) | 79.87 |  | .04 (0 ~ 4) | 81.24 |  | .08 (0 ~ 65) | 79.90 |  |
