# Supplementary material for: Typology and Impact of YouTube Videos Posted in Response to a Student Suicide Crisis: Social Media Metrics and Content Analyses
Source: JMIR Ment Health. 2021 Jun 18;8(6):e15551. doi: 10.2196/15551 (PMC8277376; doi:10.2196/15551)
Supplement: Multimedia Appendix 2 [file mental_v8i6e15551_app2.docx]

**Multimedia Appendix 2. Pairwise post-hoc tests on the relationships between different types of videos and their impacts.**

**Table 1. Pairwise comparison on the number of views between different types of video uploaders.**

|  | Top YouTubers  Standardized test statistics  (adjusted sig.) | Popular YouTubers  Standardized test statistics  (adjusted sig.) | Regular YouTubers  Standardized test statistics  (adjusted sig.) | Politicians  Standardized test statistics  (adjusted sig.) | Traditional Media  Standardized test statistics  (adjusted sig.) | Online Organizations  Standardized test statistics  (adjusted sig.) | Government bodies  Standardized test statistics  (adjusted sig.) |
| --- | --- | --- | --- | --- | --- | --- | --- |
| Top YouTubers | -- |  |  |  |  |  |  |
| Popular YouTubers | 1.600  (1.000) | -- |  |  |  |  |  |
| Regular YouTubers | 4.283  (0.000)* | 3.665  (0.005)* | -- |  |  |  |  |
| Politicians | 3.614  (0.006)* | 2.662  (0.163) | -0.521  (1.000) | -- |  |  |  |
| Traditional Media | 2.555  (0.223) | -1.098  (1.000) | 4.276  (0.000)* | 2.375  (0.368) | -- |  |  |
| Online Organizations | 3.263  (0.263) | -2.163  (0.642) | 2.457  (0.295) | 1.174  (1.000) | 1.847  (1.000) | -- |  |
| Government Bodies | 3.524  (0.009)* | 2.588  (0.203) | 0.217  (1.000) | -0.522  (1.000) | 2.218  (0.558) | 1.398  (1.000) | -- |

**p* < 0.05

**Table 2. Pairwise comparison on the number of comments between different types of video uploaders.**

|  | Top YouTubers  Standardized test statistics  (adjusted sig.) | Popular YouTubers  Standardized test statistics  (adjusted sig.) | Regular YouTubers  Standardized test statistics  (adjusted sig.) | Politicians  Standardized test statistics  (adjusted sig.) | Traditional Media  Standardized test statistics  (adjusted sig.) | Online Organizations  Standardized test statistics  (adjusted sig.) | Government bodies  Standardized test statistics  (adjusted sig.) |
| --- | --- | --- | --- | --- | --- | --- | --- |
| Top YouTubers | -- |  |  |  |  |  |  |
| Popular YouTubers | 1.317  (1.000) | -- |  |  |  |  |  |
| Regular YouTubers | 4.030  (0.001)* | 3.759  (0.004)* | -- |  |  |  |  |
| Politicians | 3.397  (0.014)* | 2.761  (0.121) | -0.496  (1.000) | -- |  |  |  |
| Traditional Media | 3.454  (0.012)* | -2.934  (0.070) | 1.531  (1.000) | 0.527  (1.000) | -- |  |  |
| Online Organizations | 4.336  (0.000)* | -4.240  (0.000)* | -0.606  (1.000) | -0.931  (1.000) | 2.305  (0.445) | -- |  |
| Government Bodies | 3.593  (0.007)* | 2.978  (0.061) | 0.593  (1.000) | -0.844  (1.000) | 1.320  (0.558) | 0.309  (1.000) | -- |

**p* < 0.05

**Table 3. Pairwise comparison on the number of comments supporting a video between different types of video uploaders.**

|  | Top YouTubers  Standardized test statistics  (adjusted sig.) | Popular YouTubers  Standardized test statistics  (adjusted sig.) | Regular YouTubers  Standardized test statistics  (adjusted sig.) | Politicians  Standardized test statistics  (adjusted sig.) | Traditional Media  Standardized test statistics  (adjusted sig.) | Online Organizations  Standardized test statistics  (adjusted sig.) | Government bodies  Standardized test statistics  (adjusted sig.) |
| --- | --- | --- | --- | --- | --- | --- | --- |
| Top YouTubers | -- |  |  |  |  |  |  |
| Popular YouTubers | 3.635  (0.006)* | -- |  |  |  |  |  |
| Regular YouTubers | 6.257  (0.000)* | 3.214  (0.028)* | -- |  |  |  |  |
| Politicians | 5.606  (0.000)* | 2.537  (0.235) | -0.198  (1.000) | -- |  |  |  |
| Traditional Media | 6.268  (0.000)* | -3.192  (0.030)* | 0.228  (1.000) | -0.051  (1.000) | -- |  |  |
| Online Organizations | 6.406  (0.000)* | -3.411  (0.014)* | -0.189  (1.000) | -0.334  (1.000) | 0.452  (1.000) | -- |  |
| Government Bodies | 5.198  (0.000)* | 2.440  (0.308) | 0.386  (1.000) | -0.471  (1.000) | 0.500  (0.558) | 0.300  (1.000) | -- |

**p* < 0.05

**Table 4. Pairwise comparison on the number of comments criticizing a video between different types of video uploaders.**

|  | Top YouTubers  Standardized test statistics  (adjusted sig.) | Popular YouTubers  Standardized test statistics  (adjusted sig.) | Regular YouTubers  Standardized test statistics  (adjusted sig.) | Politicians  Standardized test statistics  (adjusted sig.) | Traditional Media  Standardized test statistics  (adjusted sig.) | Online Organizations  Standardized test statistics  (adjusted sig.) | Government bodies  Standardized test statistics  (adjusted sig.) |
| --- | --- | --- | --- | --- | --- | --- | --- |
| Top YouTubers | -- |  |  |  |  |  |  |
| Popular YouTubers | 3.971  (0.002)* | -- |  |  |  |  |  |
| Regular YouTubers | 5.517  (0.000)* | 1.569  (1.000) | -- |  |  |  |  |
| Politicians | 5.228  (0.000)* | 1.563  (1.000) | 0.318  (1.000) | -- |  |  |  |
| Traditional Media | 5.959  (0.000)* | 2.150  (0.663) | -0.822  (1.000) | -0.229  (1.000) | -- |  |  |
| Online Organizations | 5.588  (0.000)* | -1.623  (1.000) | -0.026  (1.000) | 0.309  (1.000) | -0.848  (1.000) | -- |  |
| Government Bodies | 4.854  (0.000)* | 1.656  (1.000) | 0.720  (1.000) | -0.447  (1.000) | 0.352  (1.000) | 0.717  (1.000) | -- |

**p* < 0.05

**Table 5. Pairwise comparison on the number of comments supporting help seeking between different types of video uploaders.**

|  | Top YouTubers  Standardized test statistics  (adjusted sig.) | Popular YouTubers  Standardized test statistics  (adjusted sig.) | Regular YouTubers  Standardized test statistics  (adjusted sig.) | Politicians  Standardized test statistics  (adjusted sig.) | Traditional Media  Standardized test statistics  (adjusted sig.) | Online Organizations  Standardized test statistics  (adjusted sig.) | Government bodies  Standardized test statistics  (adjusted sig.) |
| --- | --- | --- | --- | --- | --- | --- | --- |
| Top YouTubers | -- |  |  |  |  |  |  |
| Popular YouTubers | 3.457  (0.011)* | -- |  |  |  |  |  |
| Regular YouTubers | 7.123  (0.000)* | 4.780  (0.000)* | -- |  |  |  |  |
| Politicians | 6.682  (0.000)* | 4.233  (0.000)* | 0.294  (1.000) | -- |  |  |  |
| Traditional Media | 7.040  (0.000)* | -4.655  (0.000)* | 0.488  (1.000) | 0.640  (1.000) | -- |  |  |
| Online Organizations | 7.387  (0.000)* | -5.173  (0.000)* | -0.434  (1.000) | 0.000  (1.000) | 1.000  (1.000) | -- |  |
| Government Bodies | 5.751  (0.000)* | 3.309  (0.020)* | 0.206  (1.000) | 0.000  (1.000) | 0.438  (1.000) | 0.000  (1.000) | -- |

**p* < 0.05

**Table 6. Pairwise comparison on the number of comments criticizing help seeking between different types of video uploaders.**

|  | Top YouTubers  Standardized test statistics  (adjusted sig.) | Popular YouTubers  Standardized test statistics  (adjusted sig.) | Regular YouTubers  Standardized test statistics  (adjusted sig.) | Politicians  Standardized test statistics  (adjusted sig.) | Traditional Media  Standardized test statistics  (adjusted sig.) | Online Organizations  Standardized test statistics  (adjusted sig.) | Government bodies  Standardized test statistics  (adjusted sig.) |
| --- | --- | --- | --- | --- | --- | --- | --- |
| Top YouTubers | -- |  |  |  |  |  |  |
| Popular YouTubers | 2.619  (0.185) | -- |  |  |  |  |  |
| Regular YouTubers | 4.758  (0.000)* | 2.683  (0.153) | -- |  |  |  |  |
| Politicians | 4.350  (0.000)* | 2.247  (0.517) | 0.000  (1.000) | -- |  |  |  |
| Traditional Media | 4.476  (0.000)* | -2.239  (0.528) | 0.865  (1.000) | 0.590  (1.000) | -- |  |  |
| Online Organizations | 4.599  (0.000)* | -2.434  (0.313) | 0.483  (1.000) | 0.336  (1.000) | 0.389  (1.000) | -- |  |
| Government Bodies | 3.744  (0.004)* | 1.757  (1.000) | 0.000  (1.000) | 0.000  (1.000) | 0.404  (1.000) | 0.232  (1.000) | -- |

**p* < 0.05

**Table 7. Pairwise comparison on the number of comments disclosing suicide risk between different types of video uploaders.**

|  | Top YouTubers  Standardized test statistics  (adjusted sig.) | Popular YouTubers  Standardized test statistics  (adjusted sig.) | Regular YouTubers  Standardized test statistics  (adjusted sig.) | Politicians  Standardized test statistics  (adjusted sig.) | Traditional Media  Standardized test statistics  (adjusted sig.) | Online Organizations  Standardized test statistics  (adjusted sig.) | Government bodies  Standardized test statistics  (adjusted sig.) |
| --- | --- | --- | --- | --- | --- | --- | --- |
| Top YouTubers | -- |  |  |  |  |  |  |
| Popular YouTubers | 3.838  (0.003)* | -- |  |  |  |  |  |
| Regular YouTubers | 6.764  (0.000)* | 3.624  (0.006)* | -- |  |  |  |  |
| Politicians | 6.352  (0.000)* | 3.263  (0.023)* | 0.292  (1.000) | -- |  |  |  |
| Traditional Media | 6.467  (0.000)* | -3.143  (0.035)* | 0.980  (1.000) | 0.974  (1.000) | -- |  |  |
| Online Organizations | 6.881  (0.000)* | -3.777  (0.003)* | -0.105  (1.000) | 0.226  (1.000) | 1.163  (1.000) | -- |  |
| Government Bodies | 5.467  (0.000)* | 2.551  (0.226) | 0.204  (1.000) | 0.000  (1.000) | 0.666  (1.000) | 0.156  (1.000) | -- |

**p* < 0.05
