# Supplementary material for: Typology and Impact of YouTube Videos Posted in Response to a Student Suicide Crisis: Social Media Metrics and Content Analyses
Source: JMIR Ment Health. 2021 Jun 18;8(6):e15551. doi: 10.2196/15551 (PMC8277376; doi:10.2196/15551)
Supplement: Multimedia Appendix 3 [file mental_v8i6e15551_app3.docx]

**Multimedia Appendix 3. Comparing the top 7 impactful student suicide-related videos with their pre- and post-videos’ uploaded by the same YouTubers.**

| **YouTuber** | **No. of subscribers** | **Video Type** | **Upload date** | **No. of Views** | **No. of First Layer Comments** | | **No. of Replies** | | **% of Users commented to suicide video but not pre- and post- videos** | **No. Of Characters Per First Layer Comment** | | | **No. Of Characters Per Reply** | | |
| --- | --- | --- | --- | --- | --- | --- | --- | --- | --- | --- | --- | --- | --- | --- | --- |
|  |  |  |  |  | From viewers | From uploader | From viewers | From uploader |  | Mean | SD | T-tests  (*P*-value) | Mean | SD | T-tests  (*P*-value) |
| Top 1 | 511,397 | Suicide | 11/3/2016 | 131,596 | 912 | 0 | 660 | 10 | 94.6% | 52.3 | 98.8 |  | 30.7 | 55.9 |  |
|  |  | Pre- | 10/3/2016 | 444,480 | 172 | 1 | 79 | 0 |  | 13.0 | 14.1 | < .001 | 8.9 | 7.9 | < .001 |
|  |  | Post- | 11/3/2016 | 270,784 | 254 | 1 | 79 | 1 |  | 12.6 | 12.2 | < .001 | 12.2 | 14.3 | < .001 |
| Top 2 | 533,819 | Suicide | 10/3/2016 | 121,719 | 855 | 0 | 404 | 0 | 92.2% | 39.4 | 99.3 |  | 25.3 | 41.0 |  |
|  |  | Pre- | 10/3/2016 | 178,218 | 1785 | 0 | 161 | 0 |  | 21.4 | 24.4 | < .001 | 11.6 | 12.8 | < .001 |
|  |  | Post- | 10/3/2016 | 99,883 | 144 | 0 | 18 | 0 |  | 12.7 | 12.4 | < .001 | 10.3 | 9.7 | < .001 |
| Top 3 | 115,790 | Suicide | 10/3/2016 | 72,711 | 400 | 1 | 270 | 0 | 96.8% | 49.8 | 75.7 |  | 37.0 | 68.0 |  |
|  |  | Pre- | 5/3/2016 | 95,804 | 107 | 0 | 49 | 0 |  | 15.6 | 14.9 | < .001 | 10.0 | 8.4 | < .001 |
|  |  | Post- | 11/3/2016 | 62,222 | 55 | 0 | 15 | 1 |  | 9.1 | 7.0 | < .001 | 14.0 | 13.8 | < .001 |
| Top 4 | 336,382 | Suicide | 10/3/2016 | 72,046 | 183 | 0 | 219 | 0 | 94.1% | 39.0 | 57.2 |  | 51.7 | 84.3 |  |
|  |  | Pre- | 2/3/2016 | 88,011 | 73 | 0 | 35 | 1 |  | 13.0 | 12.1 | < .001 | 17.9 | 15.2 | < .001 |
|  |  | Post- | 23/3/2016 | 80,766 | 86 | 0 | 33 | 0 |  | 10.8 | 10.6 | < .001 | 14.1 | 11.2 | < .001 |
| Popular 1 | 32,598 | Suicide | 13/3/2016 | 16,015 | 113 | 0 | 56 | 60 | 83.1% | 38.7 | 68.3 |  | 42.1 | 88.7 |  |
|  |  | Pre- | 2/3/2016 | 34,205 | 92 | 1 | 28 | 35 |  | 13.9 | 28.9 | < .001 | 13.5 | 12.4 | .011 |
|  |  | Post- | 28/3/2016 | 20,644 | 75 | 0 | 32 | 33 |  | 12.7 | 9.5 | < .001 | 19.3 | 13.7 | .038 |
| Popular 2 | 23,336 | Suicide | 10/3/2016 | 12,948 | 63 | 2 | 28 | 29 | 88.2% | 58.7 | 97.7 |  | 36.6 | 33.3 |  |
|  |  | Pre- | 6/3/2016 | 14,181 | 29 | 1 | 15 | 22 |  | 15.3 | 9.1 | < .001 | 21.3 | 14.4 | .010 |
|  |  | Post- | 15/3/2016 | 10,670 | 11 | 1 | 2 | 5 |  | 28.4 | 21.8 | .023 | 52.5 | 36.1 | .808 |
| Popular 3 | 14,299 | Suicide | 8/3/2016 | 5,288 | 68 | 0 | 42 | 47 | 86.1% | 179.3 | 280.3 |  | 98.5 | 204.3 |  |
|  |  | Pre- | 6/3/2016 | 7,188 | 20 | 0 | 3 | 6 |  | 40.2 | 34.4 | < .001 | 17.0 | 6.1 | .032 |
|  |  | Post- | 8/3/2016 | 5,638 | 33 | 0 | 2 | 18 |  | 34.8 | 35.1 | < .001 | 41.0 | 53.7 | .011 |
